# Bile Acids Activated Receptors Regulate Innate Immunity

**DOI:** 10.3389/fimmu.2018.01853

**Published:** 2018-08-13

**Authors:** Stefano Fiorucci, Michele Biagioli, Angela Zampella, Eleonora Distrutti

**Affiliations:** ^1^Section of Gastroenterology, Department of Surgical and Biomedical Sciences, University of Perugia, Perugia, Italy; ^2^Department of Pharmacy, University of Naples Federico II, Naples, Italy; ^3^Azienda Ospedaliera di Perugia, University of Perugia, Perugia, Italy

**Keywords:** innate immunity, bile acids, Farnesoid-X-receptor, G-protein bile acid receptor 1, intestinal microbiota

## Abstract

Once known exclusively for their role in nutrients absorption, primary bile acids, chenodeoxycholic and cholic acid, and secondary bile acids, deoxycholic and lithocholic acid, are signaling molecules, generated from cholesterol breakdown by the interaction of the host and intestinal microbiota, acting on several receptors including the G protein-coupled bile acid receptor 1 (GPBAR1 or Takeda G-protein receptor 5) and the Farnesoid-X-Receptor (FXR). Both receptors are placed at the interface of the host immune system with the intestinal microbiota and are highly represented in cells of innate immunity such as intestinal and liver macrophages, dendritic cells and natural killer T cells. Here, we review how GPBAR1 and FXR modulate the intestinal and liver innate immune system and contribute to the maintenance of a *tolerogenic* phenotype in entero-hepatic tissues, and how regulation of innate immunity might help to explain beneficial effects exerted by GPBAR1 and FXR ligands in immune and metabolic disorders.

Regulation of intestinal and liver immune cells reactivity against the enormous variety of antigens continuously delivered to the host by nutrients and intestinal microbiota has been the matter of extensive investigations for many years. At the steady state, the intestinal immune cells, as well as liver-resident macrophages (Kupffer cells), are settled to a state of immune tolerance, i.e., a state of unresponsiveness to tissues and bacterial antigens (1–3). While the mechanisms that support the development of this state of *anergy* are only partially defined, a growing body of evidence suggests that bile acids contribute to educate intestinal and liver immune cells (4, 5). Bile acids are a peculiar family of steroidal molecules generated by the coordinated cooperation between the host and its intestinal microbiota. Indeed, primary bile acids are generated in the liver from cholesterol side-chain breakdown and then transported through the biliary system, and released into the intestine under nutrients flow. In the intestine, primary bile acids are processed by the microbiota to generate an array of steroidal molecules known as secondary (or degenerated) bile acids [reviewed in Ref. (6)]. All bile acid species are signaling molecules, exerting pleiotropic activities in metabolic and non-metabolic tissues by activating a family of evolutionary conserved receptors collectively known as bile acids activated receptors (BARs) (6). Here, we review how bile acids signal to the host immune system and how this pathway could be exploited for the treatment of intestinal, liver, and systemic disorders.

## Bile Acid Metabolism

**Figure 1:**
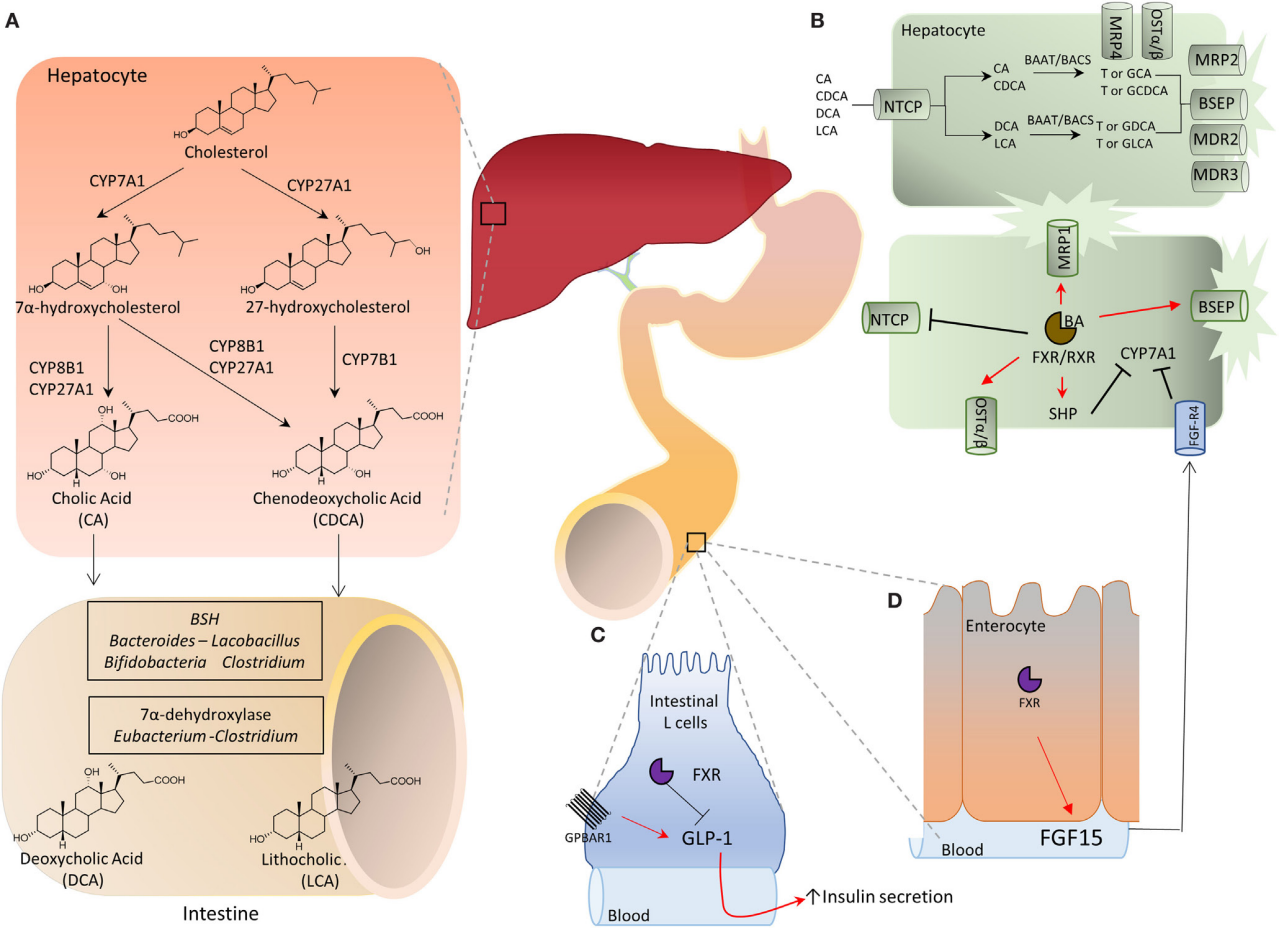
**(A)** Bile acids are synthesized in the liver from cholesterol by two metabolic pathways known as the neutral (or classical) and the acidic (alternative) pathways. In the classical pathway, cholesterol is metabolized to 7α-hydroxycholesterol by cholesterol 7α-hydroxylase (CYP7A1) and then to cholic acid (CA) by sterol 12α-hydroxylase (CYP8A1) or to chenodeoxycholic acid (CDCA) by mitochondrial sterol 27-hydroxylase (CYP27A1). On the other hand, in the acidic pathway, CYP27A1 converts cholesterol into 27-hydroxycholesterol which is then metabolized by 25-hydroxycholesterol 7α-hydroxylase (CYP7B1) into CDCA. The two primary bile acids, CA and CDCA, are then secreted into bile ducts and transported to the intestine where they are respectively converted by microbial bile salt hydrolases, an enzyme expressed predominantly by *Bacteroides, Clostridium, Lactobacillus*, and *Bifidobacteria*, and by a bacterial 7α-dehydroxylase, mainly expressed by *Clostridium* and *Eubacterium*, in deoxycholic acid (DCA) and in lithocholic acid (LCA) called secondary bile acids. **(B)** Bile acid metabolism in liver cells and adaptive changes activated in cholestasis by Farnesoid-X-Receptor (FXR). Na^+^-taurocholate cotransporting polypeptide (NTCP) is a basolateral transporter that allow uptake of bile acids (CA, CDCA, DCA, and LCA) by hepatocytes. Primary and secondary bile acids are then amidated with glycine or taurine by bile acid-CoA:amino acid *N*-acyltransferase (BAAT) or bile acyl CoA synthetase and then secreted again into bile ducts through the apical transporters bile salt export pump (BSEP), multidrug resistance-associated protein 2, and multidrug resistance protein 2 and 3. Alternatively, an outflow occurs *via* the basolateral transporters MRP4 and organic solute transporter α/β (OST α/β). The main regulatory mechanism in this pathway is contributed by the FXR which, once activated by its endogenous ligand CDCA, represses the transcription of CYP7A1 through the small heterodimer partner (SHP), thus inhibiting the classical pathway of bile acids synthesis. Furthermore, the activation of FXR up-regulates the transporters OSTα/β, MRP1, and BSEP, and down-regulates NTCP, promoting the bile acids export. **(C,D)** Activation of intestinal FXR on enterocytes causes the release of fibroblasts growth factor (FGF)-15 (FGF-19 in humans) which binds to the FGF receptor 4 (FGF-R4) on hepatocytes and inhibits CYP7A1 transcription. Further on, in the intestine secondary bile acids cause a G-protein bile acid receptor 1-dependent release of glucagon-like peptide 1 (GLP-1) from intestinal “L” endocrine cells. GLP-1, in its turn, regulates insulin secretion by pancreatic β-cells.

Once known exclusively for their ability to promote the absorption of dietary lipids (i.e., lipids, cholesterol, and fat-soluble vitamins) by the small intestine, bile acids are now recognized to function as regulatory molecules (4–6). Structurally, all mammalian bile acids are steroids and share a C_24_-5β-cholanoic acid scaffold (Figure 1A). Similarly to other steroidal hormones (i.e., gluco- and mineral-corticoids, estrogens, androgens, etc.), bile acids are generated from cholesterol breakdown. However, in contrast to these “canonical” hormones, bile acids can not be recycled back to cholesterol and, therefore, represent the endproduct of cholesterol metabolism. This pathway account for ~50% of the daily turnover of cholesterol, but only ≈200 mg of cholesterol could be excreted daily with feces as bile acids. In mammalian livers, the conversion of cholesterol in the two primary bile acids, 3α,7α,12α-trihydroxy-5β-cholan-24-oic acid (cholic acid, CA), a tri-hydroxylated bile acid, and 3α,7α-dihydroxy-5β-cholan-24-oic acid (chenodeoxycholic acid, CDCA), a di-hydroxylated bile acid, depends on two metabolic pathways known as the *neutral* (or classical) and the *acidic* (or alternative) pathway (5, 6). In the human liver, the neutral pathway accounts for the production of ~90% of primary bile acids and produces almost equal amounts of CA and CDCA. The first, and rate-limiting, enzyme in the neutral pathway is the cholesterol 7α-hydroxylase (CYP7A1) that converts the cholesterol into 7α-hydroxycholesterol (6). This enzymatic reaction is not reversible and cholesterol metabolism can only progress further to primary bile acids. Because of its critical role in bile acid synthesis, the activity of CYP7A1 is tightly regulated by a sophisticated feedback system that senses intracellular bile acids concentrations giving rise to positive and negative intra-hepatic feedback signals (4–6). The main regulatory mechanism in this pathway is contributed by the Farnesoid-X-Receptor (FXR, NR1H4), a nuclear receptor that functions as bile acid sensor in hepatocytes. Once activated by CDCA, its endogenous ligand, FXR represses the transcription of CYP7A1 mRNA by a plurality of mechanisms (Figure 1). Under binding by CDCA, FXR complexes with the Retinoid-X-Receptor (RXR), and the resulting heterodimer binds to specific responsive elements in the promoter of target genes (6). In liver cells, the recruitment of the FXR/RXR heterodimer to the SHP (small heterodimer partner, NR0B2) promoter causes the transcription of this regulatory factor (6). SHP is an atypical nuclear receptor that lacks the DNA binding domain and functions as a corepressor in the regulation of several genes, including CYP7A1 (Figure 1). In addition, activation of intestinal FXR by CDCA in enterocytes causes the release of the fibroblasts growth factor (FGF)-15 (FGF-19 in humans) which circulates back to the liver, binds to the FGF receptor 4 (FGF-R4) on hepatocytes and inhibits CYP7A1 transcription (6). In the classical pathway, the synthesis of CA proceed through a further hydroxylation at C-12 position and the above step is mediated by sterol 12α-hydroxylase (CYP8B1), and without this enzyme only CDCA (i.e., a di-hydroxylated bile acid) is formed (Figure 1). The second pathway, acidic or “alternative” route of bile acid synthesis, generates only CDCA and uses either cholesterol or oxysterols as substrates, depending on their availability in hepatocytes (6). The acidic pathway is initiated by a mitochondrial sterol 27-hydroxylase (CYP27A1) which performs the hydroxylation at C-27 position on cholesterol side chain, followed by hydroxylation on ring B (CYP7B1) and side chain shortening (CYP27A1) and contributes only 10% of CDCA generated in the liver in adulthood. However, activity of CYP27A1 increases significantly in cholestatic disorders such as primary biliary cholangitis (PBC) and might contribute to generation of CDCA.

The two primary bile acids, CA and CDCA, are secreted into bile ducts by the activity of “apical” transporters (Figure 1B), such as the ATP binding cassette subfamily B, member 11 (ABCB11) also known as bile salt export pump [BSEP] and members of multidrug resistance-associated protein (MRP) family encoded by ATP binding cassette subfamily B member (ABCC) 2, i.e., MRP2, or members of multidrug resistance protein (MDR) 2 and 3, encoded by ATP binding cassette subfamily B member 2 and 3 (ABCB), or in the blood stream by “basolateral” transporters such as organic solute transporter alpha and beta (Ostα and β) and MRP3 and MRP4 (Figure 1). This process requires their preliminary amidation, i.e., the conjugation with glycine or taurine, at the C-24 carboxyl group. This step is regulated by the bile acyl CoA synthetase and bile acid-CoA amino acid N-acyltransferase (BAAT) for tauro- and glycine-conjugation, respectively. In the human liver, CA and CDCA are amidated with glycine and taurine at a ratio ≈3:1. These give rise to “conjugated” primary bile acids, i.e., glyco-CA (GCA) and glyco-CDCA (GCDCA), and tauro-CA (TCA) and tauro-CDCA (TCDCA). In contrast to human, in mice, the large majority of bile acids (>95%) are tauro-conjugated. Importantly, while amidation with glycine or taurine changes the physio-chemical properties of CA and CDCA (essentially increases their hydrosolubility), it has no effect on affinity of bile acids toward target receptors.

**Figure 2:**
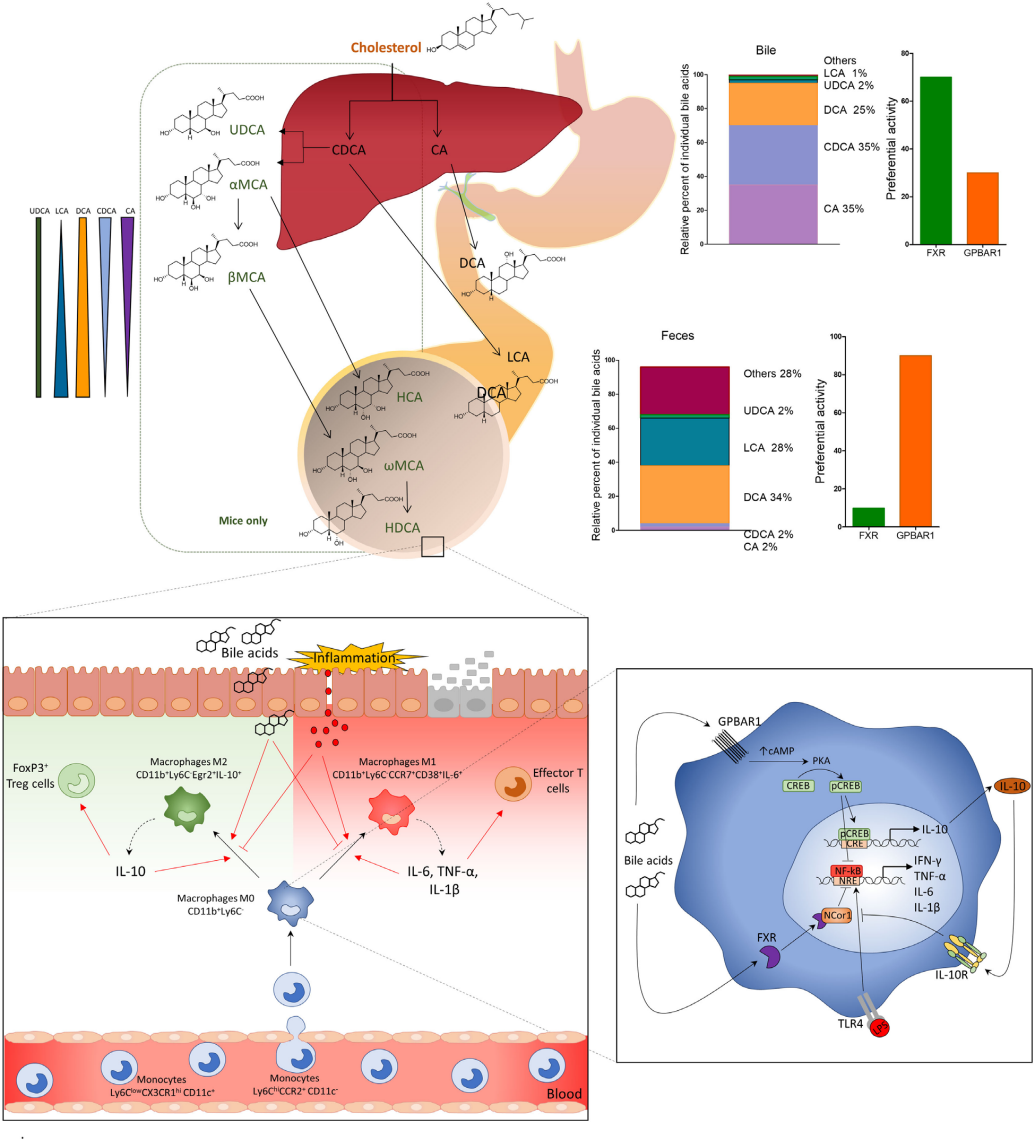
Primary bile acids (cholic acid and chenodeoxycholic acid), conjugated with glycine or taurine, are secreted in the small intestine and subjected to deamidation by intestinal microbiota *and* to 7-dihydroxylation to secondary (or degenerated) bile acids deoxycholic acid and lithocholic acid. In the distal ileum and colon, primary and secondary bile acids regulate leukocyte trafficking and macrophages differentiation *via* activation of G-protein bile acid receptor 1 (GPBAR1) and Farnesoid-X-Receptor (FXR). After breach of the epithelial barrier, or pathogenic invasion, molecules like LPS activate macrophages by TLR, thus inducing the production of pro-inflammatory cytokines, IFN-γ, TNF-α, IL-6, and IL-1β, which polarize M0 macrophages CD11b^+^Ly6C^−^ toward a pro-inflammatory M1 phenotype, CD11b^+^Ly6C^−^CD38^+^IL-6^+^. M1 macrophages might induce production of effector T cells and up-regulation of the expression of chemokine CCL2 in the colon, which attract more monocytes Ly6C^hi^CCR2^+^ from the blood to the *lamina propria*. Activation of GPBAR1 and FXR reverses this inflammatory pathway, shifts the macrophage polarization toward an anti-inflammatory phenotype M2 CD11b^+^Ly6C^−^Egr2^+^IL-10^+^, which produces high concentrations of IL-10. IL-10, in its turn, increases the production of Treg cells FoxP3^+^ and reduces the expression of CCL2 recruiting fewer monocytes from the blood to the *lamina propria* of colon. GPBAR1 signaling involves cAMP-protein kinase A (PKA)-mediated inhibition of NF-κB, and FXR signaling leads to FXR-nuclear receptor corepressor 1-mediated repression of NF-κB-responsive elements. Bile acid stimulation of both pathways blunt NF-κB-dependent gene expression. In addition, GPBAR1 directly regulate the IL-10 expression by a cAMP/PKA/p cAMP-responsive element-binding protein pathway.

In the distal ileumm (Figure 1A) glyco- and tauro-CA and -CDCA are metabolized by the intestinal microbiota to generate secondary bile acids (5). First, deamidation on the side chain occurs, operated by microbial bile salt hydrolases (BSH), an enzyme expressed predominantly by anaerobic intestinal bacteria of the genera *Bacteroides, Clostridium, Lactobacillus, and Bifidobacteria* followed by 7α-dehydroxylation on ring B. This enzymatic reaction is contributed by a bacterial 7α-dehydroxylase mainly expressed by *Clostridium* and *Eubacterium*, which converts CA in deoxycholic acid (DCA) and CDCA in lithocholic acid (LCA). DCA and LCA are secondary (or degenerated) bile acids (Figures 1 and 2). Most LCA is excreted into feces, but small amounts are circulated back to the liver, taken up by hepatocytes, amidated, and then excreted again in the bile completing a cycle in the entero-hepatic circulation (Figure 1). Other bacteria contribute to different metabolic biotransformations: *Bacteroides, Clostridium, Escherichia, Eggerthella, Eubacterium, Peptostreptococcus*, and *Ruminococcus*, that catalyze the oxidation and epimerization of the hydroxyl groups at C3, C7, and C12; *Bacteroides, Eubacterium*, and *Lactobacillus*, might carry on bile acid esterification reactions, while *Clostridium, Fusobacterium, Peptococcus*, and *Pseudomonas*, are able to desulfating bile acids under certain settings [for review see Ref. (5)].

Species-specific differences in bile acid synthesis, transport and metabolism occur. In the mice liver, a 6β-hydroxylation or 7α-epimerization on CDCA produces α-muricholic acid (αMCA) or ursodeoxycholic acid (UDCA), respectively. Epimerization at 7α-hydroxyl group transforms αMCA in βMCA. Along with MCAs, UDCA is considered to be a primary bile acids in mice (Figures 1 and 2). In pig, CDCA is predominantly converted to hyocholic acid (HCA) in the liver through 6α-hydroxylation by CYP3A4, while in mice intestinal lumen, αMCA could be converted in HCA *via* epimerization of 6β-hydroxyl group (5, 6). In mice, MCAs produced in the liver are tauro-conjugated on the side chain and sulfated/glucoronidated on their hydroxyl groups on steroidal core by sulfotransferases (SULTs) and UDP-glucuronosyltransferases and secreted in the intestine. Here, as for human, de-conjugation occurs by BSH, followed by C-6 epimerization that transforms the 6β-OH of βMCA in the 6α-OH of ωMCA. This latter in turn is subjected to a C-7 dehydroxylation to yield HDCA.

## Bile Acids Activated Receptors

The term BARs encompasses a heterogeneous family of G-protein coupled (GPCR) and nuclear receptors activated or inhibited by bile acids, with a robust species-specificity (Table 1). At the end of last century, three independent groups [reviewed in Ref. (6)] have reported that the orphan nuclear receptor FXR was a bile acid receptor. In humans, CDCA was identified, *bonafide*, as the most potent FXR ligand (CA in mice), while secondary bile acids (DCA and LCA) are preferential ligands for the G-protein bile acid receptor 1 (GPBAR1), also known as Takeda G-protein receptor (TGR)5 and membrane-type receptor for bile acids (6, 7). DCA and LCA also activate the Pregnane-X-Receptor (PXR, NR1I2) and the vitamin D receptor (VDR, NR1I1) (6). Other bile acids such as the hyodeoxycholic acid (a secondary bile acids whose concentration increases dramatically in cholestatic patients) activates the liver-X-receptor α and β (LXRα/β, NR1H3), whose physiological ligands, in humans, are oxysterols. In mice, while CA is the most abundant FXR ligand, two primary bile acids derived from CDCA (i.e., α and βMCA) function as FXR antagonists, while UDCA (a primary bile acid in mice, but a “tertiary” bile acid found in trace in humans) is a weak agonist for GPBAR1 and considered a neutral or weak antagonist toward FXR (Table 1). In addition, bile acids modulate other receptors as described in Table 1.

**Table 1:** Bile acid-activated receptors and natural and synthetic ligands.

Receptor	Tissue distribution	Natural bile acid agonists	Natural bile acid antagonists	Synthetic ligands
**Cell membrane receptors**				
G-protein bile acid receptor 1 (Takeda G-protein receptor 5)	Ileum, macrophages, gallbladder, and adipose tissue	Lithocholic acid (LCA) > DCA > chenodeoxycholic acid (CDCA) > ursodeoxycholic acid > CA		BAR501, BAR502, INT-767, and INT-777
Sphingosine-1-phosphate receptor 2 (S1PR2)	Hepatocytes	LCA		
Muscarinic receptor M2 and M3	CNS, smooth muscle cells	DCA-LCA		
fMPL	Macrophages		CDCA	
VEGF-R	Gastric and colon cancer cell lines	CDCA		

**Nuclear receptors**				
Farnesoid-X-Receptor (NR1H4)	Hepatocytes, small intestine	CDCA > CA > LCA > DCA	6βMCA	GW4064, Obeticholic acid (6-ECDCA), BAR502, Fexaramine, Px-104, tropifexor (LJN452), and LMB763
LXR	Hepatocytes	Hyo-DCA		
CAR (NR1H3)	Hepatocytes			
Vitamin D receptor (NR1H1)	Ileum, macrophages, endocrine tissues, and skin	LCA (DCA)		
Pregnane-X-Receptor (NR1H2)	Hepatocytes	CDCA-LCA (DCA)		

## GPBAR1 and FXR

While GPBAR1 and FXR are highly expressed by intestinal epithelial cells, liver parenchymal cells, the hepatocytes, only express FXR. Both receptors are also found in non-epithelial cells, including intestinal muscles and neurons (GPBAR1), biliary cells (FXR and GPBAR1), liver sinusoidal cells, and intestinal and liver endothelial cells (FXR and GPBAR1) (5). Furthermore, both receptors, as well as VDR and LXRs, are highly represented in immune cells, monocytes and macrophages, dendritic cells (DCs) and natural killer (NK) (ILC1) and NKT cells, and to lesser extent in T and B cells (7–10). While, it is unclear whether the low level expression of GPBAR1 and FXR in T and B cells results in any functional activity or is regulated in pathological conditions, activation of BARs in macrophages, DCs, and NKT, results in several regulatory functions that are inhibitory in nature, and both receptors appear to contribute to maintain the *tolerogenic* state of the liver and intestine innate immunity in face of a continuous flow of dietary xenobiotics and antigens generated by the intestinal microbiota (6). The role of the various bile acid species and their receptors in tuning intestinal immunity is not completely understood, but involves overlapping and separate mechanisms (11, 12). Support for this view comes from the observation that intestinal inflammation develops spontaneously, at the steady state, in *Fxr*^−/−^ mice with functional Gpbar1 (8, 9), as well as in *Gpbar1*^−/−^ mice (10) with functional Fxr.

It is noteworthy that by modulating the composition of bile acid pool in the intestine, the intestinal microbiota contributes to establish an *oro-aboral* gradient in the relative concentrations of different bile acid species (13). Thus, the net effect of the 7α-dehydroxylation operated by the intestinal microbiota is to decrease the amount of those bile acids, CA and CDCA, that are, by large extent, FXR preferential agonists and are most abundant in the upper intestine; while increases the concentrations of those bile acids, DCA and LCA, that are preferential agonists for GPBAR1 and are most abundant in the terminal ileum and colon (Figures 1 and 2) (6, 7). By modulating the relative abundance of different bile acids, the intestinal microbiota contributes to regulate metabolic axes and production of entero-endocrine factors such as glucagon-like peptide (GLP)-1 by the host. GLP-1 is an *insulinotropic* peptide released from intestinal “L” cells, an endocrine cell type that is most abundant in terminal ileum (14). Bile acids regulate GLP-1 secretion from L cells (Figure 1), however, *in vivo* and *in vitro* studies have shown that while FXR agonists inhibit GLP-1 secretion, the opposite is observed with GPBAR1 ligands (14), highlighting the functional relevance of different bile acids generated by intestinal microbiota for the regulation of host metabolism.

The activity of the intestinal microbiota provides an evolutionary conserved mechanism for selective and site-specific activation of FXR and GPBAR1 in different areas of the gastrointestinal tract, as confirmed by data obtained in germ-free mice (15, 16). Thus, mice raised in a germ-free condition show several abnormalities in bile acid metabolism, including a substantial increase of bile acid pool. This change appears to be driven essentially by an increased bile acid uptake in the colon in front of continuous flow of *de novo* bile acid synthesis (15, 16). Thus, despite an expansion of bile acid pool should result in a feedback repression of *de novo* synthesis of bile acids, this loop appears to be largely un-effective in mice lacking the intestinal microbiota (15, 16). The interruption of the intestinal-liver feedback has been explained with the substantial increase of the concentrations of Tα- and -βMCA and UDCA in germ-free mice (15, 16). Since Tα- and -βMCA and UDCA are primary bile acids in mice, an increase in their concentrations in the absence of intestinal microbiota (i.e., in the absence of 7α-dehydroxylation) provides a solid argument in favor of the concept that the intestinal microbial community is the engine that generates secondary bile acids. Importantly, however, as mentioned above, Tα- and -βMCA are FXR antagonists (15), while UDCA is a weak GPBAR1 agonist (Figure 1). Thus, the germ-free condition shifts the composition of bile acid pool toward those bile acids that are either FXR antagonists or GPBAR1 agonists. The resulting phenotype is biased toward a “pro-GPBAR1 phenotype,” as confirmed by increased levels of circulating GLP-1 and increased gallbladder volume (14–16). The translation relevance of these models to human physiology, however, is likely to be limited, because, MCAs are not found in humans. Nevertheless, these studies suggest that, at least in mice “the gut microbiota not only regulates secondary bile acid metabolism but also inhibits bile acid synthesis in the liver by alleviating FXR inhibition [by] the ileum” (15).

## Immune-Regulatory Effects of Bile Acids

As described previously, receptors for bile acids are expressed in several cells of innate immunity, and genetic and pharmacological studies have revealed that they participate in the fine tuning of these cells reactivity in response to bacterial and endogenous antigens. The mechanisms that mediate these effects are several and will be discussed in the following paragraphs.

### FXR and GPBAR1 and Myeloid Cells: NF-KB-Dependent and -Independent Mechanisms

Regulation of monocytes and macrophages effector functions by BARs has been demonstrated originally by Kawamata et al. (17) for GPBAR1 and by this laboratory for FXR (8, 9). Both GPBAR1 and FXR are expressed by circulating monocytes and macrophages isolated from intestine and liver (7–10, 17), while GPBAR1 is the dominant receptor expressed by liver-resident macrophages (Kupffer cells) (18). Activation of both receptors in human and rodent macrophages effectively blunt the pro-inflammatory activity of these cells, and genetic studies have shown that FXR is required for the Toll-like receptor 9-dependent inhibition of pro-inflammatory responses of intestinal macrophages elicited by CpG, a TLR-9 ligand (7, 19, 20). Several mechanisms support these inhibitory effects. We have shown that *trans*-repression of inflammatory genes caused by FXR ligands in macrophages involves a sophisticated mechanism that implies both SHP-dependent and -independent mechanisms (8, 19, 20). Indeed, the promoters of several pro-inflammatory genes, such as iNOS, TNF-α, and IL-1β are marked in the basal state by the presence of nuclear receptor corepressor 1 (NCoR1) and NCoR1-containing complexes linked to NF-κB-responsive element. This complex prevents the direct binding of κB subunit to the promoter of target genes. Activation of TLR-4 results in clearance of NCoR1 from the promoters of these genes, allowing a switch from active repression to transcriptional activation (8). As an example, under agonist binding, FXR is recruited to the iNOS and IL-1β promoters and stabilizes the NCoR1 complexes on the promoters of these two genes (9, 10). NCoR1 stabilization is essential for FXR-mediated iNOS and IL-1β *trans*-repression caused by the semisynthetic bile acid INT-747 (also known as obeticholic acid) as demonstrated by knocking down experiments with anti-NCoR siRNA (9, 10). This pathway is initiated by a ligand-induced sumoylation of FXR and mutation of the K277, located in the sumoylation domain of the receptor, to arginine (K277R) resulted in a protein that lacks the sumoylation activity and is unable to *trans*-repress TNF-α (9, 10). These regulatory effects of FXR on NF-κB and AP1 are thought to contribute to some of the beneficial effects exerted by FXR ligands (Table 1) in different therapeutic areas (Figure 2).

Another important mechanism that mediates the regulatory activity of FXR is operated by SHP. SHP is an atypical nuclear receptor that lacks the DNA binding domain and is regulated by FXR in a promoter-dependent manner (21). Because SHP is directly regulated by FXR, its expression is generally used as a readout for FXR activation. As a general mode of action, SHP functions as a corepressor forming protein–protein complexes facilitating the recruitment of corepressors at the promoter of FXR target genes (5). In 2004, we have shown that SHP physically interacts with the c-Jun subunit of AP1, thus preventing its binding to inflammatory genes (22). More recently, Yang et al. (23) have shown that SHP binds to the promoter of the CCl2, stabilizing an inhibitory complex on the promoter of this chemokine that prevents the recruitment of NF-kB subunit p65, thus repressing the expression of this potent chemoattractant. Supporting a mechanistic role for SHP in regulating inflammation downstream to FXR, it has been shown that SHP inhibits lncRNA H19, and that SHP degradation by Bcl2 increases H19 expression in models of cholestasis (24). Because H19 overexpression increases the expression of pro-inflammatory mediators, such as CD31cd1, IL-4, and IL-17 producing CD41 and CD81 cells in the liver and spleen, these findings highlight a role for SHP in regulating immune responses, at least in setting of cholestasis (25).

In addition to FXR, GPBAR1 ligands, DCA and LCA (7, 17) and other GPBAR1 selective agonists such as INT-777 and BAR501 (Table 1), counteract the activation of spleen and intestinal macrophages caused by TLR-4, by a mechanism that implies a cAMP-dependent activation of protein kinase A (PKA) and cAMP-responsive element-binding protein (CREB) (26–29). The cAMP–PKA–CREB pathway was shown to reduce NF-κB activity by a STAT1-dependent mechanism (26, 27). In addition, Pols et al. (28) and Perino et al. (29) have provided evidence that activation of GPBAR1 reduces the accumulation and activation of macrophages in aortic plaques and adipose tissues by NF-κB-independent mechanisms. Indeed, feeding *Gpbar1*-deficient bone marrow chimeric mice and myeloid cell-specific *Gpbar1*-knockout mice with a high fat diet exacerbated insulin resistance and inflammation of adipose tissues. Using these genetic models, it was shown that selective GPBAR1 gene ablation in macrophages predisposes to development of a pro-inflammatory phenotype. These effects have been linked to an impaired activation of a multi-protein aggregate formed by AKT–mTOR complex 1 (AKT–mTORC1) and translation of the liver inhibitory protein isoform of the transcription factor CCAAT/enhancer binding protein β (29). Overall, these studies confirm that, similarly to FXR, GPBAR1 functions as a braking receptor in macrophages and highlight the potential of GPBAR1 ligands in the treatment of inflammation in the context of altered metabolic states (30).

### GPBAR1 and FXR and DCs and NKT Cells

**Figure 3:**
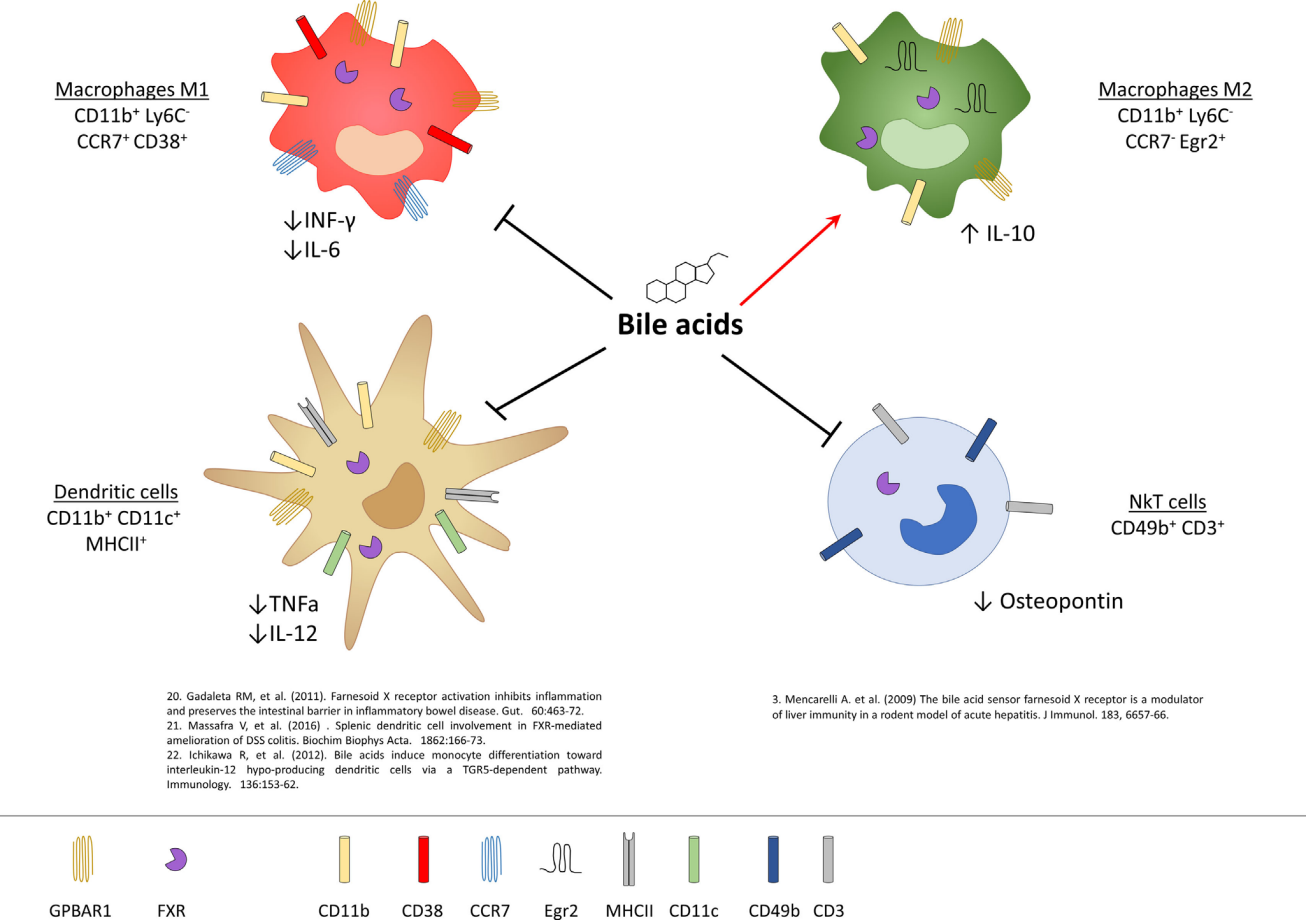
Expression of G-protein bile acid receptor 1 (GPBAR1) and Farnesoid-X-Receptor (FXR) in cells of innate immunity and their functional role. Macrophages and dendritic cells (DCs) express both GPBAR1 and FXR receptors, while for NKT cells there is currently only evidence of the expression of FXR. In macrophages, activation of these receptors by bile acids induces a polarization toward the anti-inflammatory M2 phenotype with an upregulation of IL-10 and a downregulation of the pro-inflammatory cytokines IL-6 and INF-γ. Bile acids, on the other hand, act on the DCs down-regulating the production of TNF-α and IL-12, while in NKT cells they decrease the expression of osteopontin.

In addition to monocytes/macrophages, expression of GPBAR1 and FXR has been detected in DCs and NKT cells (Figure 3) (31–33). Two studies have reported that activation of FXR by INT-747 and obeticholic acid (i.e., the same compound, see Table 1) reduces the differentiation and activation of intestinal DCs as measured by reduced ability of DCs to produce TNF-α in rodent models of colitis. Further, exposure of INT-747/obeticholic acid greatly attenuates the differentiation CD14^+^ monocytes into mature DCs (31). Confirming previous findings (8), these studies have shown that activation of FXR rescues mice from intestinal inflammation and suggested that in addition to macrophages this regulatory effect extends to other cells of innate immunity such as DCs. Similar data were reported by Massafra et al. (32), that have observed a reduced number of activated DCs in the colon of colitic mice administered with INT-747/obeticholic acid (32). In contrast, Ichikawa et al. (33) have shown that peripheral blood monocytes-derived DCs cultured with bile acids produce low levels of IL-12 and TNF-α in response to a challenge with bacterial endotoxin, but this effect appears to be FXR-independent, since the IL-12 hypo-producing phenotype could not be induced by exposing the cells to GW4064, a highly selective FXR ligand. Part of these discrepancies could be interpreted taking into account that INT-747/obeticholic acid is a dual FXR/GPBAR1 ligand (Table 1).

Liver NKT cells also express a functional FXR and activation of FXR in these cells results in a profound inhibition of their ability to produce osteopontin (see below), a potent pro-inflammatory mediator, along with IL-1β and IFN-γ (9). *In vivo*, in a rodent model of acute liver injury, activation of FXR rescues mice from acute hepatitis induced by concanavalin A, and this protective effect is associated with a negative regulation of NKT influx into the liver and negative regulation of markers of NKT activity, including osteopontin (9).

### GPBAR1 and FXR Are Negative Regulator of NLPR3 Inflammasome

**Figure 4:**
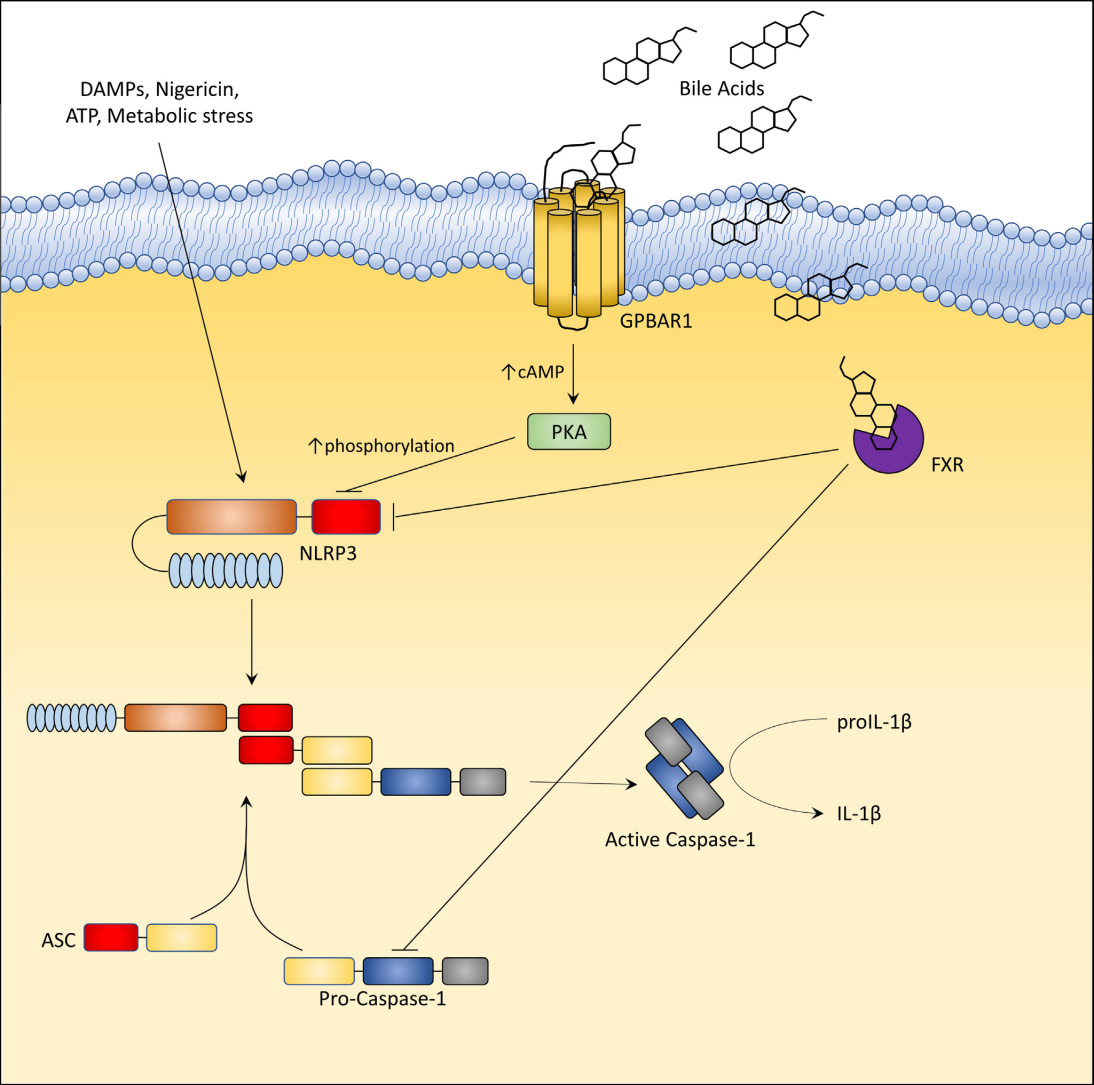
G-protein bile acid receptor 1 (GPBAR1) and Farnesoid-X-Receptor (FXR) activation inhibit the activation of NLRP3 inflammasome. In liver macrophages, GPBAR1 ligation lead to a protein kinase A (PKA)-dependent phosphorylation of NLRP3 at Ser291 (Ser295 in humans). This phosphorylation, induced by PKA kinase activation, promotes NLRP3 ubiquitination thus preventing the assembly of NLRP3 inflammasome. PKA-induced phosphorylation of NLRP3 served as a critical brake on NLRP3 inflammasome activation. Instead, FXR-mediated inhibition of the NLPR3 inflammasome appears to be mediated by the physical interaction of FXR with NLRP3 and caspase 1, thus preventing their assembly into inflammasome. However, in cholestasis, a disorder characterized by impaired secretion of bile and high levels of circulating bile acids (up to 500 µM), bile acids might activate NLRP3 inflammasome acting as DAMPs or through GPBAR1-dependent activation of the GPBAR1/EGFR-ERK/AKT/JNK pathway.

Inflammasomes (34), including NLRP1, NLRP3, NLRC4, and AIM2 family members, are a class of cytoplasmic multi-protein complexes that sense endogenous and exogenous pathogen-associated or danger-associated molecular patterns (PAMPs and DAMPs) (34). The canonical inflammasomes are made up by a nucleotide-binding domain and leucine-rich repeat-containing proteins (NLRs) or AIM2, adaptor protein ASC, and caspase-1, a protease that mediates the cleavage of precursors of cytokines of IL-1 family, i.e., IL-1β and IL-18 (34). NLRP3, one of the most comprehensively characterized inflammasomes is activated by a broad array of stimuli ranging from monosodium urate, calcium pyrophosphate to β-amyloid and islet amyloid peptide and saturated fatty acids, and is also considered a sensor for viral DNA and RNA (34). Excessive activation of NLPR3 has been detected in different inflammatory disorders, suggesting a potential role for therapeutic approaches aimed at its inhibition (34). GPBAR1 and FXR have been found to modulate NLRP3 activation (Figure 4) (35, 36). In the intestine, secondary bile acids (DCA and LCA) function as endogenous inhibitors of NLRP3 inflammasome activation by activating GPBAR1 (35). DCA and LCA cause a GPBAR1-cAMP-PKA-dependent ubiquitination of NLRP3 thus inhibiting its activation (35, 36). However, since a residual suppression of NLRP3 is maintained by LCA even in the absence of GPBAR1, it appears that secondary bile acids also exert a GPBAR1-independent activity (LCA is a weak ligand for FXR). The net effect of bile acids on NLRP3 is likely to be context dependent. Indeed, at high cellular concentrations (≈100–500 μM), bile acids, particularly the hydrophilic secondary bile acids, might function as a DAMPs (similarly to monosodium urate) causing a calcium-dependent activation of NLRP3 inflammasome. In rodent models of cholestasis (i.e., impaired bile flow from the liver), circulating macrophages are exposed to high concentrations of bile acids which facilitate the assembly of NLRP3 but only if macrophages are pre-activated by exposure to endotoxin (34, 35). While these findings might provide an explanation for excessive inflammation observed in patients with severe cholestasis, activation of inflammasome requires very high concentrations of bile acids, which are only observed in severe cholestasis. In contrast, at physiological concentrations, bile acids function as a negative modulator of NLRP3 assembly and this effect requires a functional FXR (34, 35, 37). In this model, FXR physically interacts with NLRP3 and caspase 1, thus preventing their assembly into mitochondria. Noteworthy, these counter-regulatory signals provided by FXR, might be lost in inflammation, since exposure of isolated macrophages and intact animals to bacterial endotoxin causes a dramatic reduction of FXR gene expression, leading to a unchecked activation of NLPR3 (34–36, 38). In contrast, since the expression of GPBAR1 in macrophages is not regulated by inflammation, this pathway might represent an interesting therapeutic target to attenuate unwanted activation of NLPR3 (Figure 4).

Alternatively, SHP, a downstream target gene of FXR (see above), has been shown to prevent NLRP3 formation (39). Yang et al. (39), have shown that while overexpression of SHP competitively inhibits binding of NLRP3 to apoptosis-associated speck-like protein containing a CARD (ASC) domain, SHP deficiency results in an increased secretion of IL-1β and IL-18, and excessive pathologic responses in mouse models of kidney tubular necrosis and peritoneal gout. Notably, SHP deficiency promotes the accumulation of damaged mitochondria and a sustained interaction between NLRP3 and ASC in the endoplasmic reticulum (39).

In some settings, in addition to DCA and LCA, also CDCA causes a concentration-dependent activation of NLRP3 inflammasome and secretion of IL-1β *in vitro* in LPS-primed J774 macrophages (a murine cell line) (40). However, concentrations required are very high (50–100 µM and higher) and CDCA fails to elicit IL-1β release when administered to bone marrow-derived macrophages as well as to J774 cells and Kupffer cells without pre-incubation with LPS. The activation of NLRP3 inflammasome by CDCA in this setting is reported to be GPBAR1 dependent, and mediated by the up-regulation of EGFR-ERK/AKT/JNK signaling (40). However, not only CDCA is a very weak agonist for GPBAR1 and transactivation of EGFR by GPBAR1 has been reported only in cancer cells, i.e., in cells that express very high levels of EGFR, but, surprisingly, TCA (i.e., the physiological ligand of GPBAR1) failed to elicit mature IL-1β secretion or pro-IL-1β and NLRP3 assembly even when administered to pre-activated macrophages (40). Thus, the relevance of this mechanism to immune regulation is unclear.

## BARs in Inflammation and Immune Dysfunction

The availability mice lacking the whole-body expression of GPBAR1 (41) and FXR (42), as well as conditional knockout mice lacking the expression of the receptors in restricted cell compartment, as well as selective and non-selective agonists for the two receptors, has allowed the identification of human diseases where these receptors might have a pathogenic role.

## GPBAR1 and FXR in Intestinal Immune Disorders

Inflammatory bowel diseases (IBDs) are a family of inflammatory disorders that includes at least four different clinical entities, namely Crohn’s disease, ulcerative colitis, indeterminate colitis, and lymphocytic colitis. The pathogenesis of IBD is multifactorial. However, a general consensus exists that immune dysfunction in IBD is driven, in genetically predisposed individuals, by an aberrant immune response (innate and adaptive) against intestinal microbial antigens. Alterations of the intestinal microbiota leading to intestinal dysbiosis (43, 44), a compositional and functional alteration of the gut microbiome, mainly characterized by a diminished microbial diversity occurs typically in patients with ileal Crohn’s disease. Intestinal dysbiosis in IBD is mainly contributed by a reduction of diversity within the *Firmicutes phylum* (4, 45, 46). The *Firmicutes* member *Fecalibacterium prausnitzii* has been found to be particularly under-represented in patients with ileal Crohn (4, 45, 46). Further on, in comparison to healthy controls, Crohn’s disease patients are characterized by a relative increase in *Bacteroidetes*, including *E. coli*, and *Enterobacteriaceae (Proteobacteria phylum*) (4, 45–47). These changes might impact on bile acid metabolism, since the majority of BSH expressing bacteria are members of the *Firmicutes* phylum followed by the *Bacteroidete*s and *Actinobacteria* (4, 47). A reduction *of Firmicutes* members will therefore impairs the conversion of primary bile acids into secondary bile acids, leading to elevated levels of conjugated primary bile acids in the terminal ileum (46). These microbiota-induced changes in bile acid profiles will result in a diminished GPBAR1 activity, and, to lesser extent, will also impact on FXR activity. In mice, a reduction of BSH expressing bacteria, along with a reduced generation of secondary bile acids, will increase the relative concentrations of α and βMCA, that are FXR antagonists (Table 1).

Inflammatory bowel disease patients are also characterized by an increased excretion of 3-sulfated DCA and LCA with feces (46), suggesting that in addition to BSH-dependent deamidation, other biotransformations such as sulfatation might be impaired in IBD patients (46). Sulfatation is an important mechanism of bile acid detoxification, since this step increases bile acids hydrophilicity and facilitates excretion in feces and urine (47). However, the sulfatation of DCA and LCA impacts on their affinity toward the bile acid-activated receptors and reduces dramatically their affinity for FXR (thought that secondary bile acids are, if any, weak ligands for this nuclear receptor), but might also impact on activation of GPBAR1 (thought this is less known). In addition, a reduction of LCA might impact on activation of VDR, that is a robust anti-inflammatory receptor in macrophages (48). The clinical relevance of these changes in bile acid metabolism in IBD is poorly understood, and might simply reflect the appearance of intestinal dysbiosis. Furthermore, how these changes impact on FXR/GPBAR1 balance and on integrity of GPBAR1 and FXR regulated axes remain to be defined, taking into account that species-specificity in bile acid metabolism should be considered in a translation of experimental findings to human IBD. Nevertheless, it is interesting that probiotic administration to colitic mice effectively impacts on intestinal dysbiosis and restores the FXR–FGF15 axis (49).

Independently on how the immune system is activated, treatment of IBD is increasingly focused on identification of selective approaches that modulate leukocytes trafficking toward the intestine (50). The term “leukocyte trafficking” refers to the process of attraction of leukocytes from the systemic circulation to endothelial cells and their immigration/regression from inflamed tissues (51). Active leukocytes present in the organs during the active phase of inflammation release pro-inflammatory cytokines that positively regulate the expression of adhesion molecules on endothelial cells (52). This process drives a further recruitment of leukocytes from the blood. Recruitment of monocytes at site inflammation involves the intervention of several mediators, including pro-inflammatory cytokines, chemokines and their receptors, integrins and counter-receptors. Integrins, specifically mouse αD/β2 (CD11d), αL/β2 (CD11a), αM/β2 (CD11b), and αX/β2 (CD11c) and the human α4/β7, are adhesion receptors expressed by monocytes/macrophages, neutrophils and T cells connecting these leukocytes to their counter-receptors on endothelial cells and extracellular matrix components (fibronectin, vetronectin, collagen, fibrinogen, and laminins among others). Pro-inflammatory cytokines, including TNF-α and IL-1β, increase the expression of integrins on leukocytes and up-regulate the expression of intracellular adhesion molecules-1 (ICAM-1), vascular cell adhesion protein 1, mucosal addressin cell adhesion molecule, and E-selectin on the vascular endothelium (50–53). Leukocyte integrins play, therefore, a prominent role in intestinal inflammation and an anti-α4/β7 monoclonal antibody, vedolizumab, has recently gained approval for the treating of IBD (54).

Loss-of-function studies have demonstrated that both Fxr^−/−^ and Gpbar1^−/−^ mice are prone to develop an exaggerated inflammatory response when exposed to dextran sodium sulfate (DSS) and trinitrobenzene sulfonate (TNBS), two mouse models of intestinal damage and inflammation-driven immune dysfunction (8, 10). Thus, not only Fxr^−/−^ mice are highly biased toward the development of severe diseases when challenged with DSS or TNBS (7, 10), but spontaneously develop an altered expression of inflammatory mediators and increased intestinal permeability with age (10). In both the DSS and TNBS models, as well as human samples of Crohn’s disease and ulcerative colitis, intestinal inflammation associates with reduced expression of FXR (8). Furthermore, in comparison to naïve mice, CD11b^+^ cells (equivalent to CD14 in humans) extracted (Figure 2) from the *lamina propria* of TNBS-mice release higher amounts of NF-κB-dependent cytokines, including TNF-α, IL-6, IL-1β, and INF-γ in response to treatment with lipopolysaccharide. Treatment of wild-type mice exposed to DSS or TNBS with INT-747 (Table 1), a dual GPBAR1/FXR ligand, reduced inflammation (55) and intestinal expression of IL-1β, IL-6, and monocyte chemotractant protein 1. The mechanisms that support these immune-modulatory activities of FXR in cells of innate immunity involve both NF-KB-dependent and -independent pathways as discussed in previous paragraphs (8, 31, 32, 55).

Similar to dual FXR/GPBAR1 ligands, selective GPBAR1 ligands exert anti-adhesive effects (55), and attenuate inflammation in intestinal epithelial cell lines. *In vivo* studies have demonstrated that Gpbar1 gene ablation results in a phenotype characterized by altered molecular architecture of intestinal epithelial tight junctions, with increased expression and abnormal subcellular distribution of zonulin 1 (10) and increased intestinal permeability and susceptibility to develop severe colitis in response to DSS. In addition, naïve Gpbar1^−/−^ mice are characterized by an increased expression of signature cytokines, such as IL-1β and TNF-α. Further on, the immune-histochemical analysis of GPBAR1 expression in surgical samples obtained from Crohn’s disease patients demonstrates that GPBAR1-positive CD14^+^ cells are recruited into the mucosa of these patients and highly concentrated in granulomatous areas, strongly suggesting a role for this receptor in regulating leukocytes trafficking toward the intestine (10).

More recently, a detailed analysis of GPBAR1 activity in mouse macrophages has demonstrated that GPBAR1 activation, by the steroidal ligand BAR501 (26) (Table 1) attenuates inflammation and immune dysfunction in murine models of colitis by shifting the polarization of colonic macrophages from a M1 (CD11b^+^ Ly6C^−^ CCR7^+^ CD38^+^ IL-6^+^) phenotype, to a M2 anti-inflammatory (CD11b^+^ Ly6C^−^ CCR7^−^ Egr2^+^ IL-10^+^) phenotype. The shift was confirmed by the increased expression of specific markers for M2 phenotype such as Egr2 and c-myc, and downregulation of CD38, Gpr18, and Fpr2, which are signature genes for the M1 phenotype. Analysis of intestinal leukocytes, demonstrated that Gpbar1^−/−^ mice, at steady state, are characterized by a macrophage population that was biased toward the M1 phenotype, and responded to TNBS by developing a severe disease (26). The further characterization of leukocytes trafficking in this model demonstrated that GPBAR1 agonism reverses the effect of TNBS by decreasing both colonic and circulating monocytes/macrophages, while had no effect on the ratio of resident (non classical monocytes CD11b^+^ CCR2^−^ CX3CR1^hi^ Ly6C^low^ CD11c^+^) versus inflammatory monocytes (classical monocytes, CD11b^+^ CCR2^+^ CX3CR1^low^ Ly6C^hi^ CD11c^−^) confirming that Ly6C expression *per se* does not affect the differentiation of monocytes toward a pro- or anti-inflammatory phenotype and that the differentiation of Ly6C^+^ monocytes, after they enter the tissues, depends on the organ microenvironment (51).

*Ex vivo* studies (56) have confirmed that exposure of human and murine macrophages to GPBAR1 agonists directly reduces the expression of pro-inflammatory cytokines, such as IFN-γ, IL-1β, IL-6, and TNF-α while up-regulates the expression of anti-inflammatory cytokines, particularly IL-10 and, to lesser extent, TGF-β.

The beneficial effects exerted by GPBAR1 agonism in these models were strongly associated with increased expression of IL-10 gene transcription in the intestine and enhanced secretion of IL-10 by *lamina propria*-derived macrophages (Cd11b^+^) and were abrogated by IL-10 gene ablation (26). *Ex vivo* studies carried out using human and murine macrophages have demonstrated that GPBAR1 activation leads to a promoter-specific activation of IL-10 gene transcription. This effect is mediated by a GPBAR1-PKA-CREB dependent mechanism and involves a GPBAR1-dependent recruitment of CREB to a CRE (CREB responsive element) in the IL-10 promoter (26).

## FXR and Autoimmune Liver Diseases

Immune liver disorders are a family of autoimmune diseases involving the liver parenchymal cells and bile ducts. The clinical spectrum of these disorders ranges from autoimmune hepatitis, a chronic T cell-mediated disease, with extensive hepatocytes injury and absent/minimal bile duct involvement, to PBC and primary sclerosing cholangitis (PSC), that, in contrast, are characterized by an immune-mediated progressive bile duct injury (57). PBC is an idiopathic autoimmune chronic liver disease characterized by immune-driven biliary injury leading to progressive cholestasis, a condition of impaired bile flow (57). PBC is an autoimmune disorder that manifests in genetically predisposed individuals. Apart from HLA associations, large-scale GWAS have identified 27 non-HLA loci known to be associated with PBC susceptibility (58). Most of these candidate genes are immunity-related genes, including IL-12 and IL-12RB2, TNF-α, and IRF among others (57). Also, some of the epigenetic changes observed in PBC patients point toward a dysregulated immunity, including increasing methylation of CD40L promoter in CD4 and CD8 T cells (59), or FoxP3 in regulatory T cells (60), or INF-α in T cells (60), to mentioned just a few (61). However, the molecular pathogenesis of liver damage in PBC (and PBC) involves both a dysregulated immunity and direct injury caused by the obstructed bile flow. From an immune-genetic point of view, it is now accepted that PBC emerges from multiline age loss of tolerance to mitochondrial antigens, the main of which being the E2 component of the pyruvate dehydrogenase complex (PDC-E2), which leads to an immune-mediated attack of cholangiocytes (biliary epithelial cells) causing a progressive bile duct destruction, and ultimately leading to parenchymal cell involvement and liver cirrhosis (62). This view is strongly supported by identification of both autoreactive B cells which are the source of anti-mitochondrial antibodies, directed against the PDC-E2 antigen, along with anti-PDC-E2 specific CD4 and CD8 T cells (61, 62). In addition, other cells of innate immunity including monocytes and NK cells are involved (61). The role of innate immunity in the development of PBC, however, is likely more important in progression of liver injury rather than in explain loss of tolerance (61).

The second pathogenetic mechanism of damage in PBC (and likely in PSC) is due to the accumulation of hydrophobic bile acids in the liver and the body (63). While bile duct injury progresses, the patients develop cholestatic symptoms (i.e., pruritus and fatigue, among others). In cholestasis, bile acids contribute to liver injury by causing a direct damage of hepatocytes and cholangiocytes, and by recruiting inflammatory cells to the liver (63). These cytotoxic effects are receptor-independent and directly correlate with the hydrophobic properties of bile acid species, and require fairly high concentrations (100–500 µM). At these concentrations, hydrophobic bile acids, i.e., LCA, DCA, and CDCA (in rank of potency) cause a direct cell injury and apoptotic and/or necro-apoptotic cell death (63). A variety of biochemical mechanisms have been described over the years in association with development of hepatocyte injury in these models (63), including activation of FAS/TRAIL and mitochondrial dependent apoptosis leading to caspase 3, 8, and 9 and BAD/BAX protein recruitment, increased generation of reactive oxygen species and peroxides that, in turn, activate downstream mediators such as early growth response factor-1 (64–66). These direct cytotoxic effects on hepatocytes also result in activation of pro-inflammatory programs, leading to increased expression/activity of protein kinase C, p38 mitogen-activated protein kinase (p38 MAPK), along with p53 and NF-κB, and production of NF-κB dependent mediators, such as cytokines, chemokines, and adhesion molecules (67–72). Indeed, hepatocytes perturbation by hydrophobic bile acids increases the expression of ICAM-1 (67) and neutrophil chemoattractant chemokines, such as CxCL1 and MIP-2, along with cytokines of Th1 and Th17 signature including IL-17A and IL-23 (68–73). Further on, bile acids might directly injury the biliary epithelial cells leading to generation of IL-6 and IL-1β which further contribute to expand inflammation by recruiting Th17 cells (72). All these pro-inflammatory effects are typically receptor independent and require concentrations of bile acids that are significantly higher than that observed in the sera of PSC and PBC patients, and therefore their functional relevance to the human pathology remains controversial. In contrast to hydrophobic bile acids, UDCA and its taurine-conjugated derivative exert cytoprotective activities (63) (see below).

Several adaptive changes in bile acid synthesis and metabolism take place in cholestasis (74). Indeed, the body adapts to cholestasis by modulating the expression of bile acid transporters in the liver, intestine, and kidney (Figure 1). In the liver, these protective mechanisms reduce the “orthograde” biliary excretory routes (that is blocked) while increase the bile-acid phase I and II detoxification systems and the “lateral” or alternative and basolateral flow in hepatocytes. In the intestine, changes in bile acid transporters reduce bile acid uptake, while in the kidney increases the elimination of toxic bile acid constituents from the body (74). Most of these changes are orchestrated by activation of the bile acid sensor FXR (74). The functional effects of FXR activation in cholestasis can be summarized as follow (Figure 1B): (1) reduction of endogenous bile acid synthesis by hepatocytes. As mentioned previously, expression of CYP7A1 is transcriptionally regulated by FXR, and activation of this nuclear receptor represses the activity of the enzyme by activating SHP- and FGF-15-dependent mechanisms, thus inhibiting bile acid synthesis (75, 76). (2) Reduction of bile acid uptake by the liver and intestine. These effects are mediated by the repression of Na+-taurocholate cotransporting polypeptide, OATP1, and OATP4 which mediate bile acid uptake at the basolateral membrane of hepatocytes and intestine, thus reducing bile acid uptake and interrupting their entero-hepatic circulation (74). (3) Increased liver detoxification capability. This effect is mediated by induction of bile acid sulfatation and glucuronization enzymes, such as CYP3A4, UGT2Bs, and SULT2A1 (74). (4) Increased excretion of bile acids by hepatocytes and kidney. This effect is supported by stimulation of apical (“canalicular”) transporters (BSEP, MDR2, MDr3, and BSEP) and alternative basolateral efflux transporters (MRP3, MRP4, and OSTα/β) in hepatocytes (77–79) and MRP2, MRP4, OSTα/β on the basolateral surface of renal tubular cells in the kidney (74). (5) Increased expression of nuclear receptors involved in bile acid synthesis and detoxification such as PXR (80). However, it should be taken into consideration that FXR is a negative regulator of MRP4 and CAR, at least in mice (81).

At least three different bile acids have shown effective in slowing liver disease progression in PBC (82, 83). In addition to UDCA (84, 85), obeticholic acid (86) and NorUDCA (87) exert beneficial effects on biomarkers of cholestasis, and UDCA is effective in slowing progression and need for hepatic transplant in these patients (83). The mechanisms involved in liver protection exerted by these agents in PBC are poorly defined (88). However, UDCA (84, 85), and likely NorUDCA (87), exerts some immunomodulatory activities (83), but the mechanisms supporting these effects are poorly understood, taking into consideration that both agents are inactive toward FXR and GPBAR1 (Table 1) (89). However, indirect effects on FXR or GPBAR1, due to changes in bile acid pools cannot be excluded (83, 84). In contrast, obeticholic acid is a potent FXR agonist (and to some extent GPBAR1 agonist). Obeticholic acid, a semi-synthetic bile acid, was originally described as the 6-ethyl derivative of CDCA and characterized pharmacologically in our laboratory (22, 90–92) and over the years was shown to exert immune-modulatory and anti-fibrotic effects in rodent models of inflammation (22, 91, 92) but also to increase bile flow in models of cholestasis (93) by increasing the liver expression of bile acid transporters, such as BSEP and Ostα/β. The mechanisms that support its beneficial effects in PBC could be several, and might include regulation of liver immune system. Unfortunately the use of obeticholic acid in PBC associates with a number of side effects, pruritus being the most severe. Additionally, obeticholic acid treatment carries on a significant risk for hepatic decompensation and death when administered to cirrhotic patients at the dose of 5 mg/day. These clusters of events have caused a warning by FDA and EMEA (93).

Thought that human and animal data in these setting are lacking. Partially supporting a role of immune modulation of FXR ligand in liver injury, we and other have shown that, in addition to monocytes and macrophages (see above), NKT cells (and DC) express FXR and activation of this receptor attenuates liver injury. In a model of acute hepatitis, activation of FXR reverses the liver injury caused by exposure of mice to concanavalin A (9). The finding that, compared to wild-type mice, Fxr^−/−^ mice develop a lethal hepatitis characterized by a dramatic increase in the liver expression of osteopontin, a NKT-derived factor, strongly supports a role for FXR in regulating liver NKT cells (9). *In vitro* studies have shown that FXR ligation attenuates activation of NKT cell lines (9), further suggesting a role for FXR in directly regulating these cell subtypes. Further studies are needed to determine whether FXR and peraphs GPBAr1 ligands might exert immune-modulatory effects in autoimmune liver disease.

## FXR and GPBAR1 in Metabolic Liver Disorders

Excessive deposition of lipids in the liver, due to excessive caloric intake in individual with no risk for excessive alcohol consumption, leads to progressive hepatocytes injury and represents the cause of fatty liver disease, also known as non-alcoholic fatty liver disease (NAFLD) (94). NAFLD is a highly prevalent human disorder that exists in two main clinical subtypes: non-alcoholic fatty liver (NAFL) and non-alcoholic steatohepatitis (NASH) (94). FXR and GPBAR1 ligands might exert beneficial effects in NASH by activating multiple mechanisms, including regulation of liver immune system in addition to lipid and glucose metabolism (6, 95) and ongoing clinical trials suggest that obeticholic acid (96, 97) as well as PX104 also known as GS-9674 (98, 99), a non-steroidal FXR ligand, exert beneficial effects in NASH. The mechanisms that support these beneficial effects might involve modulation of innate immunity (100) in the liver and adipose tissues as suggested by animal studies (see above). In a mouse model of NAFLD (101), treatment of obese *db/db* mice with a dual GPBAR1/FXR agonist (INT-767) has been reported to improve liver histology and to increase markers of M2 phenotype, such as CD206, Retnl a, and Clec7a, as well as the proportion of intrahepatic monocytes bearing an anti-inflammatory phenotype, i.e., Ly6C (low). *In vitro* treatment of monocytes with INT-767 led to decreased Ly6C expression and increased IL-10 production through a cAMP-dependent pathway (101). In addition, Carino et al. (102) have shown that attenuation of liver fat deposition in rodents model of NASH involves reduction of markers of liver inflammation. Thus, treating mice with BAR501, a GPBAR1 selective ligand (102), or BAR502, a dual FXR and GPBAR1 ligand (103), reverses liver steatosis and fibrosis scores along with markers of inflammation and shifts macrophages polarization from a M1 phenotype toward a M2 phenotype. Other dual FXR and GPBAR1 ligands have shown recently the same effects (104). Whether modulation of liver immune system, add to the metabolic effects of FXR ligands in clinical settings needs further investigations.

There is evidence that intestinal dysbiosis might be a causative factor in NASH (105). Investigations in patients and rodent models of NASH have demonstrate associations between intestinal dysbiosis and NAFLD, while the link with NASH is less clear (105). Recent reports suggest that NASH patients might develop a signature microbiota and that changes in the intestinal microbiota might predict the severity of liver fibrosis (106, 107). Further on, probiotic and fecal microbiota transplantation shows beneficial effects in NASH (108). Whether deleterious effects of dysbiosis or its reversal in NASH impact on bile acid metabolism and contribute to development of liver inflammation in this setting, it is not clearly understood at the moment. However, it is well known that diet-induced obesity causes a state sub-clinical inflammation and increases intestinal permeability (5), suggesting that FXR and GPBAR1 ligands might exert their beneficial effects not only by targeting lipid and glucose metabolism but also by reversing the state of inflammation at the gut–liver interface. Further studies are needed.

## GPBAR1, FXR, and Systemic Immunity

In addition to the above-mentioned effects, dual and selective GPBAR1/FXR agonists effectively reverse inflammation in mouse models of vascular inflammation and autoimmune encephalitis (EAE) (109–111). In these studies, amelioration of vascular inflammation and EAE clinical score correlates with reduced expression of molecules that are signature markers of monocyte and microglial activation, leading to a reduced trafficking of monocytes and T cells to the CNS. These studies further demonstrated that GPBAR1 and FXR agonism counter-regulate myeloid cell activation and production of inflammatory mediators including cytokines and chemokines and expression of molecules required for leukocyte trafficking such as CCR7 through the blood–brain barrier.

Similar beneficial effects related to activation of FXR and GPBAR1 have been shown in rodent models of kidney inflammation and fibrosis. In this model, obeticholic acid and INT-767, two dual GPBAR1 and FXR agonists, as well as the selective GPBAR1 agonist, INT-767, attenuate immune dysfunction-induced fibrosis by multiple mechanisms (112, 113).

The pharmacological exploitation of these pathways might allow the targeting of complex disorders to which metabolic and immune dysregulations are contributing factors, including IBD, PBC, and PSC and metabolic disorders including NAFLD/NASH, and perhaps obesity and diabetes, among others (Figure 2).

## Concluding Remarks

Bile acids are synthesized in the liver from cholesterol breakdown and further metabolized by the intestinal microbiota to generate a family of steroidal hormones that exert a wide array of regulatory functions. Similar to other steroidal hormones (i.e., glucocorticoids, estrogens, androgens, etc.), bile acids activate a family of receptors collectively known as BARs. The BARs include GPCR and nuclear receptors mainly expressed in entero-hepatic tissues. This tissue distribution allows them to efficiently couple with their ligands, i.e., primary and secondary bile acids, which are also highly concentrated in the liver and intestine. However, bile acids are also found in the systemic circulation and their receptors are expressed outside the gastrointestinal tract, mostly on circulating monocytes and tissue-resident macrophages. Genetic and pharmacological studies have demonstrated that GPBAR1 and FXR provide counter-regulatory signals that regulate leukocytes trafficking toward the intestine and that both receptors are essential to maintain a *tolegeronic* phenotype of emigrated macrophages as demonstrated by the detailed immunological characterization of Gpbar1 and Fxr knockout mice. These mice are both biased toward a pro-inflammatory phenotype at the steady state, and are more prone than their congenic littermates to develop a severe immune dysfunction in models of inflammation. However, since both Fxr and Gpbar1^−/−^ mice are characterized by increased bile acid levels and undergo adaptive changes to cope with their altered physiology (an example is the upregulation of MRP4 in Fxr^−/−^ mice) results obtained in these genetic models should be taken cautionally and their translation into human pathologies remains poorly defined.

The mechanisms that support the effects of BARs in maintaining this anergic phenotype remain to be defined, but at least in macrophage, this activity is contributed, at least in part, by a promoter-specific induction of IL-10 due to the activation of GPBAR1-PKA-CREB pathway. In addition, inhibition of NF-kB and NLPR3 inflammasome might contribute to the widespread counter-regulatory activity exerted by these receptors in immune and epithelial cells. However, since most of the study have been carried out using synthetic BAR ligands or non-physiological concentrations of endogenous ligands, the relevance of bile acids in fine-tuning liver and intestinal innate immunity needs to be further investigated.

In summary, while several aspects of BAR physiology and pharmacology need to be further investigated, there is a robust evidence to support a role for BARs in tuning the liver and intestinal innate immunity, suggesting a potential role for BAR ligands in the treatment of clinical disorders contributed by metabolic and immune dysfunctions.

## Author Contributions

Each author has contributed equally to the writing of the article and the drawing of the figures.

## Conflict of Interest Statement

The authors declare that the research was conducted in the absence of any commercial or financial relationships that could be construed as a potential conflict of interest.
